# BRD4 Silencing Protects Angiotensin II-Induced Cardiac Hypertrophy by Inhibiting TLR4/NF-*κ*B and Activating Nrf2-HO-1 Pathways

**DOI:** 10.1155/2022/8372707

**Published:** 2022-09-19

**Authors:** Ming Fang, Jun Luo, Xi Zhu, Yingbiao Wu, Xinming Li

**Affiliations:** Department of Cardiology, Shanghai University of Medicine & Health Sciences Affiliated Zhoupu Hospital, Shanghai 201318, China

## Abstract

**Background:**

Heart failure is a critical health problem worldwide, and cardiac hypertrophy is an important characteristic of heart failure. Bromodomain-containing protein 4 (BRD4) is involved in various cellular processes, including cardiac hypertrophy. This study aimed to investigate the mechanism underlying the effects of BRD4 on cardiac hypertrophy.

**Methods:**

Rat myoblast H9c2 cells were treated with angiotensin II (Ang II) to increase the mRNA and protein expressions of BRD4. BRD4 was silenced by small interfering RNA (siRNA) in H9c2 cells. Proteins involved in Nrf2-HO-1 pathway were determined by Western blot.

**Results:**

Our data suggest that BRD4 silencing attenuated Ang II, increased the percentage of TUNEL + cells and caspase-3 activity, increased oxidative stress, and increased the expression and content of pro-inflammatory cytokines. Mechanistically, we found that BRD4 silencing enhanced the protein expressions of Nrf2 and HO-1 and inhibited the TLR4 and phosphorylation of NF-kappa B in Ang II-stimulated H9c2 cells. TLR4 overexpression attenuated cardioprotection against Ang II by BRD4 silencing, including cardiac hypertrophy, oxidative stress, and inflammatory cytokine production. Additionally, TLR4 overexpression attenuated an increase in Nrf2 and HO-1 proteins and decreased phosphorylated NF-kappa B in H9c2 cells.

**Conclusion:**

Our results speculate that the BRD4/TLR4 axis might be a promising strategy for treating cardiovascular diseases with cardiac hypertrophy, including HF.

## 1. Introduction

Heart failure is a complex clinical syndrome caused by any abnormal cardiac structure or function, which leads to decreased cardiac output, impaired ventricular filling, or increased intracardiac pressure in the state of rest or stress. It is the end stage of a variety of cardiovascular diseases. In recent years, researchers generally believe that cardiac hypertrophy is the main pathogenesis of heart failure [1]. The occurrence of cardiac hypertrophy is mostly considered to be related to neuroendocrine system dysfunction such as sympathetic nervous system excitation and renin-angiotensin-aldosterone (RAAS) system activation [2, 3]. However, after treatment with angiotensin II invertase inhibitor (ACEI), angiotensin II receptor antagonist (ARB), *β* receptor blockers, and other drugs, cardiac hypertrophy in patients with heart failure has not been significantly improved, with high mortality [4]. This also shows that the activation of neurohormonal system cannot fully explain the occurrence of cardiac hypertrophy. Therefore, it is essential to explore the detailed molecular pathogenesis of underlying cardiac hypertrophy to develop potential therapeutic strategies for heart failure.

Cardiac hypertrophy is the adaptive response of the heart to maintain cardiac function under physiological and pathological overload. It is characterized by the increase in cardiomyocyte volume and myocardial mass. At this time, the total amount of cardiomyocytes increases and the contractility increases, so that the heart can maintain normal systolic function [5]. However, this compensatory mechanism can only be maintained for a short period, and continuous hypertrophy eventually results in decompensation, heart failure, and sudden death [6]. Cardiac hypertrophy activates several fetal genes, including elevated expressions of ANP, BNP, and *β*-MHC [7]. Among signals modulating cardiac hypertrophy, the renin-angiotensin system (RAS) is essential and helps to maintain cardiovascular homeostasis. The RAS regulates blood pressure, and its activation involves atrial structural remodeling and cardiac hypertrophy of HF [8]. Angiotensin II (Ang II) is an integral part of the RAS, and it induces vasoconstriction by promoting the production of vasopressin from the central nervous system. Therefore, Ang II is a crucial stimulating agent for hypertension, heart hypertrophy, and heart failure [9, 10].

Toll-like receptor 4 (TLR4) is a receptor of the innate immune system, and it regulates chronic inflammation and produces an inflammatory factor in acute infarcted myocardium [11]. TLR4 mediates Ang II-induced cardiac hypertrophy, remodeling, fibrosis, and cardiac dysfunction [12]. In Ang II-induced hypertension rats, a blockade of TLR4 delayed the progression of hypertension and reduced the TNF-*α* and IL-1*β* levels and NF-*κ*B activity in myocardial tissue, which improved cardiac hypertrophy [13]. Moreover, TLR4 is associated with the risk and progression of HF [14]. Compared with the controls, HF patients had significantly higher activity levels of TLR4 in monocytes after acute myocardial infarction [15]. Therefore, a blockade of excessive TLR4 expression is a promising therapeutic strategy for cardiac hypertrophy in HF. However, the molecular mechanisms that modulate TLR4 in cardiac hypertrophy remain unclear.

Bromodomain-containing protein 4 (BRD4) belongs to the bromodomain and extra-terminal (BET) family and is a protein that binds acetylated lysine. A high expression of BRD4 was found during cardiac hypertrophy induced by phenylephrine and high glucose (HG) and suppressed by JQ1, which is a specific BRD4 inhibitor [16, 17]. The BRD4 expression was upregulated by a pressure overload in the endothelial cells of the mouse heart, and its functional inhibitor JQ1 attenuated the transverse aortic constriction-induced cardiac fibrosis and preserved cardiac function [18]. Moreover, BRD4 played a crucial role in the pathogenesis of elevated glucose-induced cardiomyocyte hypertrophy by the protein kinase B (AKT) pathway [17]. This study aimed to investigate whether BRD4 silencing alleviates cardiomyocyte hypertrophy, as well as whether BRD4 modulates TLR4 signaling. To establish a hypertrophy model, rat myocyte cell lines H9c2 were stimulated with Ang II. Additionally, the detailed mechanisms underlying the effects of BRD4 on hypertrophy were investigated.

## 2. Materials and Methods

### 2.1. Cell Culture and Treatment

H9c2 is a rat cardio-myoblast cell line purchased from the Cell Bank of Chinese Academy of Sciences (Shanghai, China) and cultured in high-glucose DMEM containing 10% fetal bovine serum (FBS) at 37°C in a CO_2_ incubator with a humidified atmosphere. The cells were cultured to 70%–80% confluence and underwent serum-free starvation in a DMEM overnight. Finally, they were treated with 1 *μ*M Ang II for 48 hours.

### 2.2. Small Interfering RNA (siRNA) and Exogenous Overexpression

To knockdown BRD4, H9c2 cells were transfected with a specific siRNA against BRD4 (siBRD4) (5′-GAUGAAGCCUGUAGAUGUA-3′) (GenePharma Co., Ltd., Shanghai, China). A non-targeting siRNA was used as a negative control (siNC) (5′-UUGGCAAAAGUUCUCUGCGU-3′). To overexpress TLR4, human TLR4 cDNA (MIM: 603030) was amplified and cloned into a pcDNA3.1 vector (Invitrogen, Gaithersburg, MD, USA) to construct TLR4 overexpressing plasmids (pcDNA-TLR4). The empty pcDNA3.1 vector served as a negative control (vector). The H9c2 cells were seeded into six-well plates (5 × 10^5^ cells/well) in DMEM with 10% FBS. After 24 hours, the cells in the siBRD4 group were transfected with either siBRD4 (final concentration: 100 nM) or pcDNA3.1 vectors (4 *μ*g plasmid DNA) using Lipofectamine 2000 (Life Technologies, NY, USA). After 48 hours of transfection, a qRT-PCR and Western blot were performed to measure the gene transfection efficiency.

### 2.3. Immunofluorescence Analysis of the Cell Surface Area

The H9c2 cells were fixed with 4% paraformaldehyde for 20 minutes, permeabilized in 0.5% Triton X-100 for 20 minutes, and blocked with 1% BSA for 30 minutes at room temperature. Then, the cells were incubated with a primary antibody against *α*-actinin (1 : 200, diluted in 1% BSA; Sigma-Aldrich) overnight at 4°C. Then, the cells were incubated with a fluorescent secondary antibody (1 : 200, Alexa Fluor 488, Invitrogen) for one hour. Finally, the samples were stained with DAPI (1 : 1000, Sigma-Aldrich) to mark the nucleus and observed under a fluorescence microscope (Olympus, BX60, Japan). The cell surface area in the H9c2 cells was analyzed using ImageJ software and calculated from at least 100 randomly chosen cells in each group.

### 2.4. TUNEL Staining

The cells were incubated with 4% paraformaldehyde in PBS for one hour at room temperature. After washing with PBS, the samples were blocked with 3% H_2_O_2_ for 10 minutes at room temperature and permeabilized with 0.1% Triton X-100 for 2 minutes on ice. After washing twice with PBS, the cells were incubated with a 50 *μ*L TUNEL reaction mixture for one hour at 37°C. The samples were then stained with DAPI solution for 30 minutes at room temperature to mark the nucleus. Finally, the cells were observed under a fluorescence microscope (515–565 nm; Olympus, BX60, Japan). The number of TUNEL + cells was counted.

### 2.5. Caspase-3 Activity

The caspase-3 activity was assessed using a colorimetric caspase-3 assay kit (BioVision, Inc.). The H9c2 cells were lysed to obtain the supernatant by centrifugation at 10000 g for 10 minutes at 4°C. The supernatant (30 *μ*L) was then mixed with caspase-3 substrate Ac-DEVD-pNA (10 *μ*L, 200 *μ*M) at 37°C for two hours, and a microplate reader measured the absorbance at 405 nm. The caspase-3 activity in each group was normalized to that of the control group.

### 2.6. Evaluation of Oxidative Stress

The H9c2 cells were harvested and lysed to obtain the supernatant by centrifugation. The supernatant was incubated with a detection working solution for MDA (Cat. No. A003-1; Nanjing Jiancheng Bioengineering Institute, Nanjing, China) or SOD (Cat. No. A001-1) at 37°C for 15 minutes. Then, the MDA level and the SOD activity were evaluated by measuring the absorbance values at 532 nm (MDA) or 520 nm (SOD) using a microplate reader. The MDA and SOD data were expressed as mmol/mg protein and U/mg protein, respectively.

### 2.7. ELISA

ELISA was performed to measure the inflammatory cytokines in the supernatant. The culture media were collected, and the ELISA kits determined IL-1*β*, IL-6, and IL-10. A microplate reader measured the absorbance at 450 nm. IL-1*β*, IL-6, and IL-10 were calculated based on the standard curve and expressed as pg/mL.

### 2.8. Real-Time *PCR*

The total RNA was extracted using a TRIzol reagent (Invitrogen) and reversely transcribed into cDNA using Oligo (dT) and reverse transcriptase (Takara Bio, Inc., Otsu, Japan). A quantitative PCR was carried out by SYBR Premix Ex Taq (Takara Bio) using Applied Biosystems 7500 Fast Real-Time PCR System (Applied Biosystems; Foster City, CA, USA). The sequences of the PCR primers are shown in Table 1. The PCR conditions were set as follows: initial denaturation at 94°C for 5 minutes, followed by 40 cycles at 94°C for 15 seconds and 58°C for 30 seconds. The 2^−ΔΔCt^ method was used to determine the relative levels of mRNA expression.

### 2.9. Western Blot

The proteins were extracted from H9c2 cells, and the protein samples (50 *μ*g) were separated on 10% SDS-PAGE gels and transferred to PVDF membranes. The membranes were blocked with 5% low-fat milk in Tris-buffered saline containing 0.05% Tween-20 (TTBS) and incubated with the primary antibody against TLR4 (1 : 1000; sc-293072, Santa Cruz), Nrf2 (1 : 500; ab92946, Abcam), NF-*κ*B p65 (1 : 500) and p-NF-*κ*Bp65 (1 : 500; sc-8008, Santa Cruz), and p-NF-*κ*B p65 (1 : 500; sc-166748, Santa Cruz) overnight at 4°C. The *β*-actin and lamin B were used as internal control for total and nuclear proteins. The membranes were then incubated with a horseradish peroxidase-linked secondary antibody (1 : 1000) for one hour at room temperature. The proteins were detected using a chemiluminescent detection system (Thermo Scientific, Waltham, MA, USA) and exposed to the film. The density of the bands was analyzed using ImageJ software.

### 2.10. Statistical Analysis

The data were expressed with mean ± standard deviation (SD) and analyzed using SPSS 20.0 software (SPSS, Inc., Chicago, IL, USA). The normality of the data was performed using the Kolmogorov–Smirnov test. A one-way ANOVA was used for comparisons of multiple groups, followed by the Bonferroni test. *P* < 0.05 was considered statistically significant.

## 3. Results

### 3.1. BRD4 Silencing Inhibited Ang II-Induced Hypertrophic Responses in Cardiomyocytes

**Figure 1:**
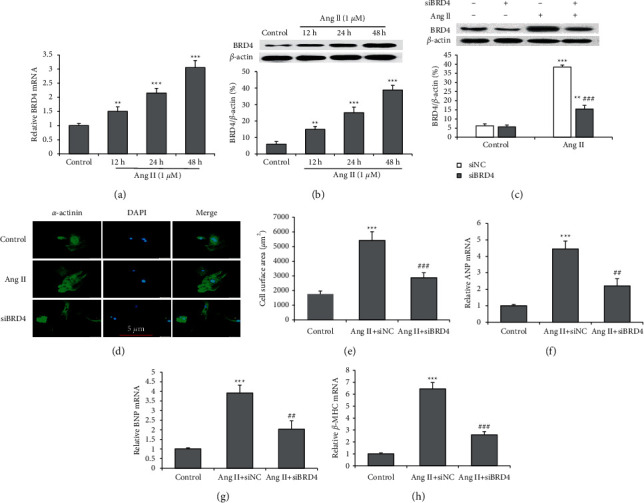
siBRD4 inhibits Ang II-induced cardiomyocyte hypertrophy. Cultured H9c2 cells were treated by vehicle (control) or Ang II (1 *μ*M) for 12, 24, and 48 hours. (a) The mRNA expression level of BRD4 in Ang II-induced H9c2 cells. (b) The protein expression level of BRD4 in Ang II-induced H9c2 cells. (c) The BRD4 expression was silenced using siRNA in H9c2 cells and validated using a Western blot. (d) H9c2 cells were stained with *α*-actinin (green) and DAPI (blue) to show morphology and size; representative photographs are shown (scale bar: 10 *μ*m). (e) Cardiomyocyte hypertrophy was evaluated by the surface area. mRNA expressions of the ANP (f), BNP (g), and *β*-MHC (h) genes were evaluated using a real‐time PCR. All data are expressed as mean ± SD, and the experiment was performed three times, ^*∗∗*^*P* < 0.01, ^*∗∗∗*^*P* < 0.001 vs. control group; ^###^*P* < 0.001 vs. Ang II + siNC group.

H9c2 cells were treated with Ang II to evaluate the expression of BRD4. Ang II treatment at 12, 24, and 48 hours markedly increased the mRNA and protein expressions of BRD4 in H9c2 cells (Figures 1(a) and 1(b)). We then performed loss-of-function experiments using siRNAs, specifically targeting BRD4 in H9c2 cells. The Western blot showed that the BRD4 protein expression was reduced in the cells transfected with BRD4 siRNA (siBRD4) compared with the cells transfected with the control siRNA (siNC) after Ang II incubation for 48 hours (Figure 1(c)). To explore the function of BRD4 in cardiac hypertrophy, immunofluorescence was carried out using antibodies of *α*-actinin. The results showed that the H9c2 cell treatment with siBRD4 had a suppressive hypertrophic response to Ang II, with significantly reduced cardiomyocyte size (cell surface area) (Figures 1(d) and 1(e)). The mRNA expressions of cardiomyocyte hypertrophy markers, including ANP (NPPA), BNP (NPPB), and *β*-MHC (MYH7), were increased in Ang II-treated H9c2 cells. However, siBRD4 markedly reversed the Ang II-induced hypertrophic responses (Figures 1(f)–1(h)).

### 3.2. BRD4 Silencing Protected H9c2 Cells from Ang II-Induced Apoptosis

**Figure 2:**
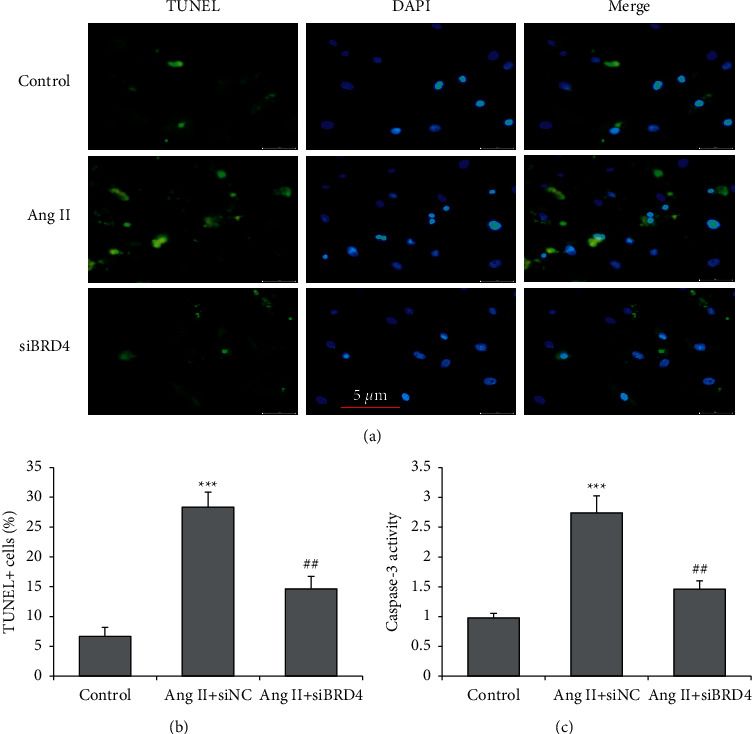
siBRD4 protects H9c2 cells from Ang II-induced apoptosis. H9c2 cells were transfected with siBRD4 or siNC and treated with Ang II (1 *μ*M) for 48 hours. (a) H9c2 cells were stained with TUNEL to show the apoptotic cells. (b) The number of TUNEL + cells was counted. (c) The caspase-3 activity was determined. Each experiment was performed in triplicate and repeated at least three times. ^*∗∗∗*^*P* < 0.001 vs. control group; ^##^*P* < 0.01 vs. Ang II + siNC group.

To observe whether BRD4 could influence Ang II-induced apoptosis of cardiac cells, H9c2 cells were transiently transfected with siBRD4 or siNC and simultaneously treated with Ang II (1 *μ*M) for 48 hours. The TUNEL assay revealed that Ang II induced a significant reduction in the number of TUNEL-positive cells with siBRD4 (*P* < 0.001) (Figures 2(a) and 2(b)). The caspase-3 activity was further analyzed, and we found that siBRD4 attenuated the increase in caspase-3 activity by Ang II (Figure 2(c)). The results indicated that siBRD4 protected H9c2 cells against Ang II-induced apoptosis.

### 3.3. BRD4 Silencing Suppressed Ang II-Induced Oxidative Stress and Enhanced Nrf2 and HO-1 Expressions

**Figure 3:**
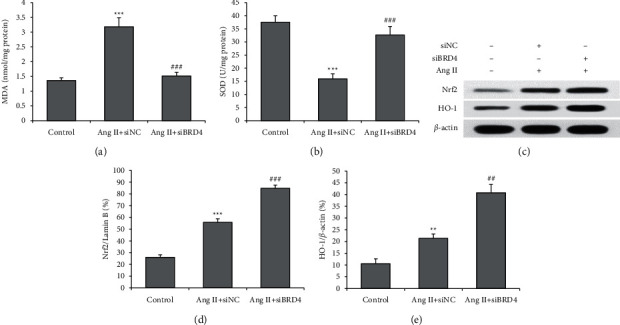
siBRD4 suppresses Ang II-induced oxidative stress in H9c2 cells by upregulating Nrf2 and HO-1. Respective commercial kits examined the MDA level (a) and SOD activity (b) in the control group, Ang II + siNC group, and Ang II + siBRD4 group. (c) The nuclear Nrf2 and total HO-1 protein levels were determined using a Western blot, and the representative protein bands are shown. siBRD4 increased the nuclear Nrf2 (d) expression and HO-1 (e) in H9c2 cells with Ang II stimulation. Each experiment was performed in triplicate and repeated at least three times. ^*∗∗*^*P* < 0.01, ^*∗∗∗*^*P* < 0.001 vs. control group; ^##^*P* < 0.01, ^###^*P* < 0.001 vs. Ang II + siNC group.

Following that, we explored whether the siBRD4 modulates Ang II-induced oxidative stress, and the MDA content and SOD activity were measured. An increase in the MDA level and a decrease in the movement of SOD were observed in the H9c2 cells following Ang II compared with the control cells. However, treatment with siBRD4 restored the increase in the MDA level and decreased the SOD activity in Ang II-stimulated H9c2 cells (Figures 3(a) and 3(b)). To explore the molecular mechanism underlying the protective effects by siBRD4, a Western blot was performed to determine the Nrf2 and HO-1 protein levels (Figure 3(c)). Ang II stimulated an increase in the expression of Nrf2 and HO-1 proteins in H9c2 cells, which was further markedly increased by siBRD4 (both *P* < 0.001) (Figures 3(d) and 3(e)). These results indicated that siBRD4 suppresses oxidative stress and activates Nrf2-HO-1 pathways in Ang II-induced H9c2 cells.

### 3.4. BRD4 Silencing Reduced the Inflammatory Response of H9c2 Cells Induced by Ang II

**Figure 4:**
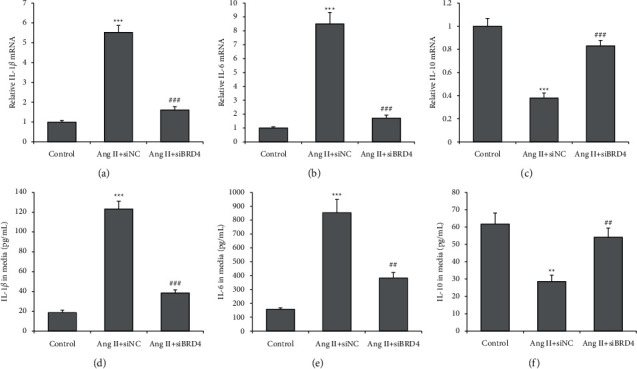
siBRD4 attenuates the Ang II-induced inflammatory response in H9c2 cells. A real-time PCR was performed to detect the mRNA levels of IL-1*β* (a), IL-6 (b), and IL-10 (c) in H9c2 cells. ELISA was performed to detect the secreted IL-1*β* (d), IL-6 (e), and IL-10 (f) in the cell supernatant of H9c2 cells. Each experiment was performed in triplicate and repeated at least three times. ^*∗∗*^*P* < 0.01, ^*∗∗∗*^*P* < 0.001 vs. control group; ^##^*P* < 0.01, ^###^*P* < 0.001 vs. Ang II + siNC group.

A real-time PCR and ELISA were performed to evaluate the expression and production of inflammatory cytokines. Ang II treatment significantly enhanced mRNA expressions of IL-1*β* (IL1B) and IL-6 (IL6) and reduced IL-10 (IL10) mRNA expression in H9c2 cells, and these changes were all reversed by siBRD4 (Figures 4(a)–4(c)). Similar results were also found in supernatant inflammatory cytokines. Compared with the Ang II group, IL-1*β* and IL-6 secretion in culture media decreased, and IL-10 secretion increased in the Ang II + siBRD4 treatment group (*P* < 0.01) (Figures 4(d)–4(f)). This result indicates that siBRD4 has significant anti-inflammatory effects in H9c2 cells with Ang II.

### 3.5. BRD4 Silencing Suppressed TLR4-NF-kB Signaling in Ang II-Induced H9c2 Cells

**Figure 5:**
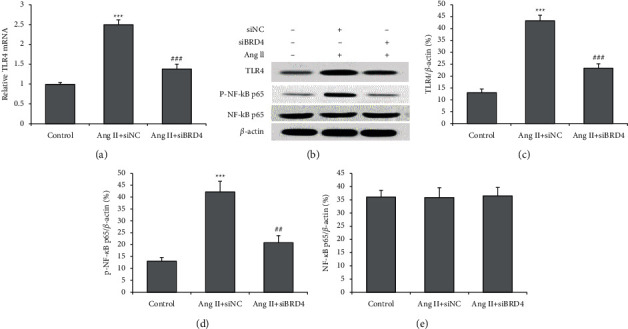
siBRD4 inhibits the TLR4-NF-*κ*B pathway in Ang II-induced H9c2 cells. Real-time PCR was performed to measure the mRNA level of TLR4 (a). Western blot was carried out to determine the TLR4 (b), phosphorylated NF-*κ*B p65(c), total NF-*κ*B p65(d), protein expression in H9c2 cells (e). Each experiment was performed in triplicate and repeated at least three times. ^*∗∗∗*^*P* < 0.001 vs. control group; ^##^*P* < 0.01, ^###^*P* < 0.001 vs. Ang II + siNC group.

The mRNA level of TLR4 was measured using a real-time PCR and showed that siBRD4 attenuated the increase in TLR4 mRNA by Ang II (Figure 5(a)). The TLR4 and NF-*κ*B p65 expressions in H9c2 cells were then determined using a Western blot (Figure 5(b)). Ang II markedly increased both TLR4 and the ratio of phosphorylated NF-kB p65 to total NF-kB p65, and this effect was reversed by siBRD4 (Figures 5(c)–5(e)). However, the total NF-kB p65 protein remained unchanged after the Ang II or siBRD4 treatment (Figure 5(e)). These findings suggest that siBRD4 suppresses Ang II-activation of TLR4-NF-kB signaling.

### 3.6. Overexpression of TLR4 Reversed the Protective Effects of siBRD4 on Ang II-Induced H9c2 Cells

**Figure 6:**
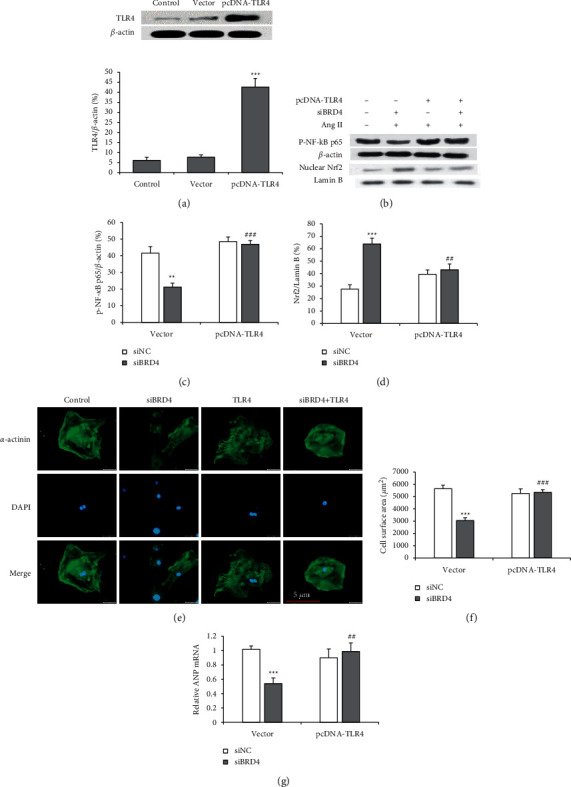
Overexpression of TLR4 reduces siBRD4-mediated protection against Ang II-induced cardiac hypertrophy. The induced Ang II cells were used as a control in the experiments. (a) A Western blot confirms the efficiency of TLR4 overexpression after 48 hours. ^*∗∗∗*^*P* < 0.001 vs. control group. (b) H9c2 cells were co-transfected with BRD4-specific siRNA (siBRD4) and pcDNA-TLR4 or vector and then subjected to Ang II for 48 hours. A Western blot was performed to determine NF-kB p65 (b), (c) and nuclear Nrf2 (d) in H9c2 cells. Immunofluorescence was performed to evaluate the hypertrophic responses (e), and the cell surface area was compared (f). The mRNA expression of ANP was detected using a real-time PCR (g). Each experiment was performed in triplicate and repeated at least three times. ^*∗∗*^*P* < 0.01, ^*∗∗∗*^*P* < 0.001 vs. vector + siNC group; ^##^*P* < 0.01, ^###^*P* < 0.001 vs. vector + siBRD4 group.

We employed a gain-of-function approach to explore the role of TLR4 in siBRD4-mediated cardioprotective effects. A pcDNA3.1 vector carrying TLR4 (pcDNA-TLR4) was constructed and transfected into H9c2 cells to upregulate the expression of TLR4 transiently. The efficiency of TLR4 overexpression was confirmed by a Western blot (Figure 6(a)). Subsequently, we investigated the effect of TLR4 overexpression on the expression of phosphorylated NF-kB p65 and Nrf2 in H9c2 cells. siBRD4 decreased phosphorylated NF-kB p65 and increased Nrf2 protein expressions in Ang II-induced cells. However, the transfection of pcDNA-TLR4, but not the empty vector, reversed these effects (Figures 6(b)–6(d)). Furthermore, the suppressive effects of siBRD4 on hypertrophic responses were attenuated by pcDNA-TLR4 (Figures 6(e)–6(g)).

### 3.7. Overexpression of TLR4 Eliminated the Effects of siBRD4 on Oxidative Stress and Inflammation

**Figure 7:**
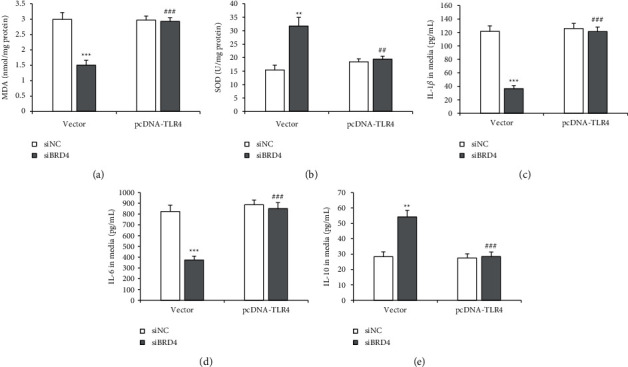
Overexpression of TLR4 attenuates the siBRD4-induced inhibition on oxidative stress and inflammatory cytokine. Ang II-induced cells were used as a control. Respective commercial kits examined the MDA level (a) and SOD activity (b) in cells of siBRD4 and/or pcDNA-TLR4. ELISA measured the secreted IL-1*β* (c), IL-6 (d), and IL-10 (e) in the cell supernatant. Each experiment was performed in triplicate and repeated at least three times. ^*∗∗*^*P* < 0.01, ^*∗∗∗*^*P* < 0.001 vs. vector + siNC group; ^##^*P* < 0.01, ^###^*P* < 0.001 vs. vector + siBRD4 group.

We investigated the effect of pcDNA-TLR4 on oxidative stress and the expression of inflammatory cytokines. pcDNA-TLR4 reversed the decrease in MDA and increased SOD by siBRD4 in H9c2 cells exposed to Ang II (Figures 7(a) and 7(b)). The content of IL-1*β* and IL-6 was significantly decreased, and the anti-inflammatory cytokine IL-10 was increased by siBRD4 in Ang II-induced H9c2 cells. Overexpression of TLR4 reversed the decrease in IL-1*β* and IL-6 and increased IL-10 by siBRD4 (Figures 7(c)–7(e)).

## 4. Discussion

In this study, we explored the effect of BRD4 in H9c2 cells stimulated with Ang II. The principal findings were that Ang II increased BRD4 expression in H9c2 cells. H9c2 cells were transfected with siNC or siBRD4. Therefore, the possibility that transfections induce changes in hypertrophy, apoptosis, oxidative stress, and inflammation was eliminated where possible (Figures 1–7). BRD4 silencing by siRNA markedly suppressed cardiac hypertrophy, apoptosis, oxidative stress, and pro-inflammatory cytokine production. In addition, BRD4 silencing enhanced the protein expressions of Nrf2 and HO-1 and suppressed the TLR4 and phosphorylation of NF-kappa B in Ang II-stimulated H9c2 cells. The cardioprotective effects of BRD4 silencing, including cardiac hypertrophy, oxidative stress, and inflammatory cytokine production, were all significantly attenuated by TLR4 overexpression. Moreover, the increase in Nrf2 and HO-1 proteins and the decrease in phosphorylated NF-kappa B by siBRD4 depended on the TLR4 in H9c2 cells. Our findings showed that BRD4 silencing has protective effects by inhibiting cardiac hypertrophy, oxidative stress, and inflammation in H9c2 cells induced by Ang II. The mechanism may be related to enhancing Nrf2 and HO-1 and inhibiting the TLR4-NF-*κ*B signaling pathway.

In our study, Ang II increased the mRNA and protein expressions of BRD4 in H9c2 cells. As Ang II also simultaneously induced cardiac hypertrophy, this indicates that BRD4 might involve the process of cardiac hypertrophy. Our results are in accordance with other reports that BRD4 expression is influenced by phenylephrine or high-glucose levels, which are two inducers of cardiac hypertrophy [16, 17]. Moreover, the hypertrophic signal-induced BRD4 expression and BRD4 were recruited to the gene encoding ANF [16, 19]. Our study showed that Ang II treatment enhanced the mRNA expressions of ANP, BNP, and *β*-MHC and supported the potential role of BRD4 in the transcription of these cardiomyocyte hypertrophy markers. BRD4 silencing by siRNA attenuated Ang II-induced hypertrophy, as evidenced by the reduced cell surface area. Additionally, BRD4 silencing attenuated the Ang II-induced increase in RNA expressions of ANP, BNP, and *β*-MHC. This showed that BRD4 is an inducer of cardiac hypertrophy, which was supported by a recent report that Ang II-induced cardiomyocyte hypertrophy was attenuated by BRD4 decrease [20]. The promotive effect of BRD4 on cardiac hypertrophy was also observed in H9c2 cells incubated with high glucose [17]. However, these markers alone do not promote hypertrophy; therefore, further research on the mechanism underlying BRD4 is required for investigation.

How BRD4 silencing attenuated oxidative stress of H9c2 cells was observed by our study. As an inducer of cardiac hypertrophy, oxidative stress promotes cardiac hypertrophy by activating various cellular signaling pathways and modulating the extracellular matrix function [21]. In pressure overload-induced cardiac hypertrophy, oxidative stress was increased by impairing the mitochondrial respiratory chain complexes [22]. A reduction in oxidative stress by BRD4 silencing might contribute to the antihypertrophic effect, which is supported by other studies that reported that some agents alleviated hypertrophy by inhibiting oxidative stress [23, 24].

This study focused on the roles and mechanism of BRD4 in cardiomyocyte hypertrophy. An in vitro model was established in H9c2 cells stimulated with Ang II. This cell model can simulate various diseases, including hypertension, heart failure, and AF. Though fibrosis seems to be a more critical hallmark of AF, this phenotype cannot be presented in H9c2 cells (cardiomyocyte). Cultured cardiac fibroblasts and animal models should be applied to simulate fibrosis in AF.

Inflammation involves many pathological processes of AF, which contribute to AF's occurrence and maintenance [25]. Additionally, systemic inflammation is associated with cardiac hypertrophy. For instance, serum inflammatory cytokines, such as TNF-*α* and IL-6, were significantly elevated and positively correlated with cardiac fibrosis and left ventricular wall thickness [26]. TNF-*α* and IL-1*β* are two cytokines produced after the triggering of inflammatory response. When macrophages are stimulated by the outside stimulator, they will produce a large number of pro-inflammatory factors and early inflammatory markers, which are secreted and released under the conditions of tissue damage, microbial infection, and immune system activation [27, 28]. There is evidence that when macrophages are stimulated by Ang II, TLR4 signaling pathway is activated to produce NF-*κ*B activation, thus increasing transcription of TNF-*α* and IL-1*β* [29, 30]. Therefore, this study used H9c2 cells to evaluate whether Ang II could induce the secretion and expression of pro-inflammatory factors through TLR4 signaling pathway and whether BRD4 silencing could play an anti-inflammatory role by inhibiting the secretion and expression of these pro-inflammatory factors. Our study showed that Ang II increased pro-inflammatory cytokine production (IL-1*β* and IL-6) and decreased anti-inflammatory cytokine production (IL-10), which was reversed by BRD4 silencing. Additionally, BRD4 silencing inhibited TLR4 and the phosphorylation of NF-kappa B in Ang II-stimulated H9c2 cells. TLR4 is a primary receptor of the innate immune system and plays a vital role in the induction of the inflammatory response in myocardial ischemia [7]. Moreover, the activation of TLR4 induces the expression of NF-*κ*B and releases many cytokines that are responsible for local and systemic inflammation, such as TNF-*α* and IL-1*β* [31]. Furthermore, TLR4 mediates Ang II-induced cardiac hypertrophy and is associated with myocardial TNF-*α* and IL-1*β* levels and NF-*κ*B activity [12, 13]. Our study showed that the mRNA and protein of TLR4 were reduced by BRD4 silencing in H9c2 cells with Ang II, and TLR4 overexpression could attenuate cardiac hypertrophy change, oxidative stress, and inflammatory cytokine production. This result suggests that BRD4 may be a potential upstream modulator of TLR4 in cardiac hypertrophy and partly mediate oxidative stress and inflammation.

Our results showed that although the protein levels of p-NF–*κ*B p65 and Nrf2 were markedly increased after Ang II induction, they showed a different response to BRD4 silencing, as p-NF-*κ*B p65 was reduced and Nrf2 was increased by siBRD4. Although increased NF-*κ*B means an increased inflammatory response in cardiomyocytes, an increased level of Nrf2 may indicate a state of cell defense for overcoming increased oxidative stress [32, 33]. Therefore, BRD4 silencing attenuated the oxidative stress and inflammatory response in the cells. Moreover, these changes modulated by siBRD4 were abolished in cells with TLR4 overexpression. This observation indicates that the decreased induction of oxidative stress and inflammation is due to the reduced activation of TLR4. TLR4/NF-*κ*B is a signaling pathway that is activated during myocardial hypertrophy [34]. This study showed a BRD4-TLR4-NF-*κ*B axis in promoting cardiac hypertrophy. This axis' detailed mechanisms should be investigated in further research, especially in in vivo animal models.

There is one major limitation in this study. Ang II-induced H9c2 cardiomyocyte is a common in vitro model to study cardiomyocyte hypertrophy. However, Ang II-induced cardiomyocyte hypertrophy might be due to the paracrine release of TGF-*β*1 and endothelin-1 from cardiac fibroblasts [35]. Therefore, our study shows that BRD4 might have a direct effect on cardiomyocyte hypertrophy, and whether BRD4 also regulates hypertrophic signal in cardiac fibroblast needs further investigation.

## 5. Conclusion

In conclusion, Ang II increases the mRNA and protein expressions of BRD4 and promotes cardiac hypertrophy, oxidative stress, and inflammatory cytokine production. Meanwhile, BRD4 silencing protects against Ang II-induced cardiac hypertrophy via the upregulation of Nrf2-HO-1 and downregulation of TLR4-NF-*κ*B signaling pathways. Therefore, our results provide important insights into the molecular mechanisms underlying the cardioprotective effects of BRD4 silencing, which may help the development of novel therapeutic strategy for BRD4 inhibitor in the treatment of cardiac hypertrophy.

## Figures and Tables

**Table 1:** List of oligonucleotide primer sequences used in this study.

Genes	PCR size	Forward primer (5′–3′)	Reverse primer (5′–3′)
BDR4	180 bp	ACAGCCCCAACAGAACAAAC	GCTGGTTCCTTCTTGCTCAC
ANP	141 bp	ACCAAGGGCTTCTTCCTCT	TTCTACCGGCATCTTCTCC
BNP	256 bp	AGAACAATCCACGATGCAGAAG	AAACAACCTCAGCCCGTCACA
*β*-MHC (MYH7)	126 bp	AAGGGCCTGAATGAGGAGTA	GGCTTGAAGGAAAATCGCTT
IL-1*β*	120 bp	GCTGTGGCAGCTACCTATGTCTTG	AGGTCGTCATCATCCCACGAG
IL-6	102 bp	AGTTGCCTTCTTGGGACTGA	ACTGGTCTGTTGTGGGTGGT
IL-10	210 bp	GCCCAGAAATCAAGGAGCA	CGTAGGCTTCTATGCAGTT
TLR4	191 bp	AAGTTATTGTGGTGGTGTCTAG	GAGGTAGGTGTTTCTGCTAAG
*β*-actin	183 bp	CCTCTATGCCAACACAGTGC	TGGAAGGTGGACAGTGAGGC

## Data Availability

The data are available upon request to the corresponding author.
